# Hospital Festivals and Meetings

**Published:** 1895-01-26

**Authors:** 


### HOSPITAL FESTIVALS AND MEETINGS.

### RADCLIFFE INFIRMARY, OXFORD.

A quarterly general court of the governors of this institution was held on Wednesday, Viscount Dillon in the chair There was a large attendance.

The Chairman presented the treasurer's financial statement. The total receipts in 1893 amounted to £7,101 7s. 5d.; in 1894 to £6,883 1s. 11d. The expenditure in 1893 was £7,680 3s. 6d.; in 1894, £7,508 6s. 7d. Comparing 1894 with 1893, £218 5s. 6d. less was received and £171 16s. 11d. less spent. The debts of the last three years made a present total indebtedness of £2,626 14s. 5d. He was of opinion that they might, if possible, transfer money from the establishment fund to the amount of £300 a year, but they were not always in a condition to do so.

Mr. Daniel, Worcester College, hoped the court would stick strongly to the conviction that the establishment fund was capital account, and was only to be dealt with as capital expenditure. The main bulk of their indebtedness had been incurred since 1890. He desired as a matter of honesty and morality, and also in order that the public should understand what their responsibility was, that they would let the deficit stand, and go out to the public.

The Master of University said the policy of having a large indebtedness seemed to be rather questionable. He thought the last argument of Mr. Daniel was hardly up to the morality which as managers of property they ought to cling to. It seemed a large question to keep legacies as capital, because it was a very doubtful thing whether a very largely-increased capital was a desirable arrangement for the hospital at all. When the establishment fund was called into existence this matter was talked over, and it was arranged that £50,000 should be looked upon as fixed and unchangeable capital; that all money beyond that should be subject to the immediate action of the court. The arrangement by which that was done was that all legacies and small donations and so on beyond a certain figure were to be brought into the establishment fund, which when it reached £500 was to be invested, the object being that when things which might be looked upon as capital charges arise there should be always something by which they could be paid, without having to administer the ordinary income of the establishment. It seemed to him that what was really intended was that the hospital should have the right of handling with a considerable amount of freedom at a general meeting such sums as were in the establishment fund, and he believed that lowering of their indebtedness was a duty they had to discharge by transferring from the establishment fund or taking a certain portion of their capital, and he therefore had much pleasure in moving that £300 be transferred from the establishment fund to the general account, to carry on the real business of the infirmary.

The Rev. C. J. H. Fletcher seconded, and said that Mr. Daniel seemed to depreciate any transfer from the establishment fund towards current expenditure, and his policy seemed to be to let a sensational, or, as he called it, a knock-me-down, deficit accumulate until it had the necessary effect on the Oxford public. If that policy would succeed in shocking the Oxford public to the extent of shaking the necessary money out of their pockets, he thought that there would be something to be said for it, but he doubted if it could be done. He thought they should follow the practice of the London hospitals in making use of legacies for ordinary expenditure. Until they could raise their subscription list he thought their policy was to make use of legacies as they came in for the purpose of paying their debts and preventing a large accumulation of debt.

In the course of the discussion a doubt was raised by several speakers as to the power of the court to transfer the sum of £300 without due notice, and ultimately the Master of University gave notice that at the next Court he would move to sell out stock to the amount of their indebtedness, viz., £2,626 14s. 5d., and the subject dropped.

#### Report of the Special Committee.

The special committee appointed to enquire into the present financial state and prospects of the infirmary, reported that the deficit of £2,001 9s. 9d. existing on December 31st, 1893, was the growth of the last three years. In the last three years there has been an average expenditure of £7,558 12s. 7d., and an increased expenditure as compared with the year 1890 of £636 6s. 11d. in 1891, £695 4s. 10d. in 1892, £660 11s. 6d. in 1893. Your committee finds that there is not any one cause to which this sudden rise of expenditure can be attributed. The annual report for 1891 speaks of a greater outlay under every head of expenditure, and suggests that attacks upon the hospital have tended to increase ordinary expenditure by giving rise to a feeling that possibly the hospital was not quite up to modern requirements Your committee has, however, noticed that in 1891 the expenditure of the year was doubtless increased by the maintenance of the fever patients. The report for 1892 records an outbreak of diphtheria, which added very largely to the expenses of the fourth quarter, disused wards having to be reopened and special assistance obtained. The report for 1893 records an increase of 24 per cent. of in-patients, and 9 per cent. of out-patients and casualties, as well as the opening of two special wards. It is also to be observed that the reports of other infirmaries show that expenditure everywhere increases with the constant improvement of hospital accommodation and treatment. While thinking that the explanations given above may to some extent account for the increase in the expenditure, your committee felt the necessity of making, with the assistance of your officers, a detailed examination of the ordinary expenditure. Careful inquiry has been made under the several headings of an exhaustive classification prepared for the purpose of this examination. Dealing with articles of consumption, surgical and medical accounts, washing and cleaning, and salaries and wages, maintenance and increase of income, your committee is firmly of opinion that if the income of the infirmary is to be increased or even maintained, a more systematic method of personal canvass for subscriptions must be adopted. The appointment of a standing sub-committee charged with the duty of maintaining an adequate subscription list, and that employment of authorised collectors, paid or unpaid, would lead to many of those whom the appeals made at present do not reach, becoming contributors to the funds. To take only the case of the City of Oxford. Although during the last ten years the population as well as the rateable value has considerably increased, the annual subscriptions (including those for children's wards and chaplain's funds) were in 1893 less by £337 17s. 4d. than in 1884. From a careful comparison of the subscription list for 1893 with the Oxford directory it appears that less than one-third of the official and professional classes, and less than one-tenth of the trading class are to be found among the supporters of the infirmary. Your committee cannot but suppose that this is due to the fact that the claims of the institution are not brought with sufficient directness to the notice of every member of these classes of the community. Economy and efficiency of expenditure. (*a*) The prices of purchases made as those of the infirmary are out of moneys subscribed by the public, must be constantly subjected to very careful examination and inquiry; (*b*) larger quantities of goods should be delivered at one time, and efficient arrangements made for their storage and issue. The disuse of the old buildings by patients may afford some opportunity for reform in this respect. There appears to be no reason why coal should not be bought at the pit mouth at the most favourable season of the year, and stored upon the premises until required. (*c*) The officers of the different departments should be encouraged to make within the limits of their office suggestions for the prevention of waste and the initiation of economy. It should be considered in this connection whether any rearrangement of the duties of the members of the staff is desirable, and in particular whether it might not be desirable to appoint, as is now done in many hospitals, a house governor or steward, who should be responsible (subject to the directions of the honorary treasurer) for all the details of administration.

The Master of University proposed a cordial vote of thanks to the committee for the great trouble they had taken in the matter, and that the report be referred to the Committee of Management, with instructions to give effect to the suggestions in it as far as they considered it possible, so to do without application to the court, and to report to the court what had been done. There was only one thing which seemed beyond the power of the committee, and that was the appointment of a new superintendent.

Dr. Collier seconded the motion, which was carried unanimously.

Mr. Daniel drew attention to the fact that in the distribution of the Hospital Sunday Fund, which amounted to £216 14s. 5d., a fund which was ostensibly for the medical charities of Oxford and the Radcliffe Infirmary, that the amount allocated to the infirmary was only £12 4s. 2d., which was the balance after certain amounts had been voted to other institutions. He complained strongly of the small amount allocated to the infirmary.

The Master of University gave notice that at the next court he would move that the infirmary after the year 1895 should be a free hospital. He said there seemed to be an idea that the infirmary was a close corporation, and the public did not grasp the fact that it was the county hospital and the largest of such establishments in the country. He believed they would be in a stronger position if the hospital were free.

The meeting then terminated.

### THE AFTER-CARE ASSOCIATION.

This association, which has for its excellent object the "after-care of poor convalescents on leaving asylums for the insane" (a title, by-the-way, which is for various reasons to be altered to one which it is thought will better express its intentions), held its annual meeting on Monday last at 83, Lancaster Gate, the house of Lord Meath, the president. The chair was taken by the Rev. Canon Elwyn, D.D., Master of the Charterhouse. Amongst others present were Dr. Hack Tuke, Dr. Rayner, the Rev. Henry Hawkins, Dr. Shuttleworth, Mr. J. P. Richards, and Mr. H. Thornhill Roxby, secretary.

Much useful work is done quietly and unostentatiously by this association, and it is worthy of every support. The sad condition of friendlessness in which too many poor patients find themselves when discharged "recovered" from asylums, especially in the case of women, is very distressing, and here it is that this association steps in and endeavours to find suitable employment for those who, once more able and anxious to do good work, find themselves heavily handicapped in the search. From the report for the past year it is seen that 113 cases (100 were women) came before the committee in 1894, most of which have been materially assisted. To secure the success of the association it is recognised that the co-operation and help of the medical superintendents of asylums and of the guardians of the poor must be obtained, and it is satisfactory to find that this co-operation is being increasingly met with.

The Rev. F. Hall, of Friern Barnet, moved the adoption of the report, and in so doing said he was most anxious to do what was possible to strengthen the hands of Mr. Hawkins, whose name was so especially associated with this society. He thought there was nothing more striking as a witness to Christianity at the present day than the way in which the principles laid down by Christ were now being carried out, more especially so in the care of the sick and of children.

Dr. Savage said he had watched the gradual development of the association, which was all the healthier perhaps for its slow growth. The public, and even members of the medical profession who had not special experience in such cases, had much to learn in regard to mental disease. They had to realise that to imagine directly a man was sane he was sound and able to battle with the world was as far from the truth as if anyone suffering from typhoid fever were to be accounted well so soon as his temperature became normal. The association did much to help those who were leaving asylums "recovered" to once more obtain positions of trust.

Mr. J. P. Richards briefly recounted some of the objects and aims of the association, speaking of the difficulty experienced in finding employment for the people taken under their protection, particularly in the case of domestic servants and governesses.

The resolution to change the name of the society to "The After-care association for poor persons discharged recovered from asylums for the insane," was proposed by Dr. Rayner and seconded by Dr. Shuttleworth, and votes of thanks were proposed by the Rev Henry Hawkins.

For the benefit of those who are interested in the association's work we have pleasure in making known that a drawing-room meeting on its behalf is to be held, by permission of Miss Phipps, at Hilhaw, Purley, Surrey, on January 18th at three o'clock p.m.

